# Enhancing father involvement of earthquake-affected fathers: a qualitative analysis

**DOI:** 10.3389/fsoc.2025.1657517

**Published:** 2025-11-28

**Authors:** Mehmet Fatih Güloğlu, Yusuf Adigüzel

**Affiliations:** 1 Departmant of Sociology, Kilis 7 Aralık University, Kilis, Türkiye; 2 Faculty of Communication, Sakarya University, Sakarya, Türkiye

**Keywords:** children, container city, earthquake, father, father involvement, phenomenology

## Abstract

This study identifies the factors influencing father involvement in container cities following the February 6, 2023 earthquakes in Türkiye and examines the current state of father involvement from a sociological perspective. Father involvement improves children’s cognitive, social and emotional development as well as academic success. However, earthquakes can seriously disturb this. Previous studies have indicated that the father-child relationship transforms after disasters such as earthquakes. This study analyzes, within “father involvement” framework, how father-child relations differs in families forced to set up new life-world in container cities. The analysis was grounded in the phenomenological sociology tradition, which interprets father involvement as a lived sociological phenomenon embedded in everyday experiences. The study utilizes Van Manen’s hermeneutic phenomenological approach. Data collected from 23 earthquake-affected fathers (*N* = 23), using a semi-structured interview, a purposive sampling technique, and analyzed using MAXQDA. The factors affecting father involvement were thematized as follows: the child’s social and psychological well-being, changes in emotional state, economic deterioration; search for meaning of life, and transformations in relationships with the spouse and the surrounding environment. Father involvement is categorized into three dimensions: responsibility, interaction, and communication. Father involvement is context-dependent, and the factors influencing it differ post-earthquake compared with the existing literature. The earthquake motivated fathers to prioritize their children, highlighting the importance of father involvement.

## 1 Introduction

The earthquakes in Türkiye on February 6, 2023, affected approximately 14 million people. Excluding those who were still missing, the earthquakes resulted in the deaths of 53,537 individuals and injured 107,204 others. As of 2024, 392 container cities have been established and these had been provided accommodation for 675,291 individuals. Specifically, 183 container cities in Hatay accommodate 205,603 people, whereas 55 container cities in Kahramanmaraş accommodate 135,215 people (Presidency of the Republic of Türkiye, Directorate of Strategy and Budget, 2024).

In 2022, of the 14,013,196 people living in the earthquake-affected region, 4,805,937 were children aged 0–17 years (Turkish Statistical Institute, 2022). This figure shows that one in every three people affected by the earthquake was a child. Approximately 120,000 children currently live in container cities in Hatay and Kahramanmaraş. Kousky (2016, p. 76) and Yılmaz (2024, p. 143–145) stated that disasters lead to both short -and long- term physical harm to children, disrupt access to education and healthcare, result in the loss of possessions or family members, lead to their participation in the post-disaster labor force, increase their vulnerability to diseases, and cause stress and trauma.

Accordingly, earthquakes affect children in diverse and complex ways. In mitigating these adverse effects, children’s secure attachment to their parents and the parents’ assumption of caregiving responsibilities play a critical role. As Juth et al. (2015, p. 1309) note, “parents and young children typically go through the post-disaster adjustment period together as an interdependent unit, or dyad, because physical proximity is required for the child to receive and the parent to provide care.” In this context, establishing a secure attachment between fathers and children is especially important to protect children from the potential harm of the disaster. The development of secure attachment may be achieved through fathers’ active involvement. Father involvement helps children better navigate the challenges they face and make sense of distressing events, while also contributing to their well-being, even in the most difficult circumstances.

This study aims to identify the factors that shape father involvement to mitigate potential risks to children’s development in the aftermath of an earthquake. Elucidating the conditions that determine post-earthquake father involvement is critical for supporting children’s developmental trajectories. This study also describes the current levels and forms of father involvement. Evidence based on how father involvement transforms after an earthquake is limited. However, there is a body of work on the post-disaster or post-traumatic transformation of relationships among family members, reinforcement of masculine identities, intergenerational transmission and effects of trauma, stress-related disorders, and family functioning (Austin, 2016; Chen et al., 2021; Giannakopoulos et al., 2025; Juth et al., 2015; Proctor et al., 2007). To the best of our knowledge, no studies have directly and explicitly examined the relationship between earthquakes and father involvement. This study addresses that gap by employing a phenomenological method grounded in the phenomenological sociological tradition, and -by foregrounding the sociological nature of the concept- offers an original contribution to the literature.

## 2 Theoretical framework: father involvement in container city

### 2.1 Father involvement

When paternal authority over family members has diminished or even dissolved (Thernborn, 2004), father involvement has ironically gained increasing significance day by day. The collective importance placed on fatherhood, driven by the awareness of continuing to be a family, compels fathers to seek respect within family members. Fathers can reproduce this respectability through the quality of their contributions to family life. The belief in the necessity of sustaining the family and the social benefits generated by family relationships legitimized the father involvement. Given the lifelong and intergenerational nature of the socialization process, the internalization of human production by individuals constitutes a sociological reality. This internalization is made possible through human beings’ *openness to the world* and their capacity as *world-constituting beings*, whose externalizations attain legitimacy within the social order (Berger and Luckmann, 2008; Güloğlu, 2019). As Berger and Luckmann (2008, p. 137) note, “legitimation justifies the institutional order by giving a normative dignity to its practical imperatives.” In this sense, father involvement become increasingly institutionalized and legitimized on a global scale. Thus, father involvement has become a central focus of social policy (Osborne, 2020).

In the context of rehabilitating child-rearing practices within the family, democratizing relationships among its members, and supporting individual agencies among family members, the concept of father involvement has garnered special attention in the field of social sciences and has also become a subject of sociological research (Güloğlu, 2021). For instance, Strier and Perez-Vaisvidovsky (2021) attempted to construct a non-hegemonic fatherhood theory within the framework of feminist theory. This is because the intergenerational transfer of social, cultural, and economic resources reflects the sociological position of fathers (Keizer, 2020, p. 48). In summary, father involvement is not merely a personal or familial matter but a sociological phenomenon.

The concept of father involvement has begun to highlight their importance in shaping a child’s life-world. Father involvement refers to the father’s active role in the upbringing and development of a child. Many theoretical approaches have been developed regarding father involvement. For instance, Pleck (2010) developed a three-factor model of father involvement: responsibility, accessibility, and engagement. Palkovitz’s model of father involvement, cognitive, affective, and behavioral factors (including emotional climate, behavioral style, and relational synchronization) are emphasized (Palkovitz, 2002; Palkovitz, 2010). Cook et al. (2005) analyzed father involvement at both emotional and instrumental levels. There are also studies that emphasize the more sociological aspects. Adamsons (2006) study, which highlights the impact of a father’s identity on father involvement, is particularly noteworthy in this regard. In his study, Adamson stated that whether the father’s identity and status are centered affects father involvement. Similarly, Cabrera et al. (2007), p. 186) developed a dynamic model of father involvement, considering family characteristics, as well as the contextual and individual traits of both fathers and children. Pleck’s (2007) comprehensive review of theories on father involvement introduced integrated concepts such as attachment theory, social capital theory, Bronfenbrenner’s ecological systems theory, and essential father theory, culminating in the proposal of “the integrated ecological-parental capital theory.” Additionally, Epstein et al. (2003) proposed a layered model of father involvement that examines the concept within the family, school, and community triangles. However, these approaches still fall short of presenting a holistic theory of father involvement.

Father involvement can be generally classified into psychology-based approaches based on father-child interactions and their outcomes (Lamb et al., 1987; Palkovitz, 1997; Pleck, 2010) and approaches that focus on individual or social factors (Cabrera et al., 2000; Palkovitz and Hull, 2018; Parke, 2000). From the perspective of contributions to children, father involvement positively influences their psychological development and educational outcomes (Erdem, 2020, p. 410; Sarkadi et al., 2008). High levels of father involvement affect children’s cognitive and language development, social, emotional, and moral growth, as well as their psychopathological development (Fitzgerald et al., 2020, p. 9). Taşkın (2011) emphasized the significant role of fathers in children’s overall development; Gözübüyük and Özbey (2020) reported that the father-child relationship positively contributes to children’s intrinsic motivation; and Tükel and Uzunöz (2020) stated that the frequency of fathers’ play with their children supports children’s social development. Fathers provide emotional support to their children and ensure their well-being and development (Cabrera et al., 2018; Lamb, 2000; Parke, 2000; Pleck, 2010). However, the subject of father involvement within the unique context of container cities has not yet been explored. Before addressing father involvement in container cities, it is essential to identify and clarify the factors that influence father involvement.

### 2.2 Factors influencing father involvement

Several factors affect father involvement including: ethnicity, economic capital, social class, the father’s level of education, investments made in the fatherhood identity, child-related characteristics, the father’s socio-cultural environment, and the academic self-esteem of both parents (Gracia, 2014; Lamb, 2000; Özcan and Aydoğan, 2014; Pekel Uludağlı, 2017; Yoleri, 2022). Additionally, co-residence with the child and the child’s gender also affects father involvement (Lundberg et al., 2007, p. 91). In addition, the father’s character, mother’s influence, and the relationship between spouses also determine father involvement (De Santis and Barham, 2017, p. 960). Furthermore, family type, the social, cultural, and economic capital of both the father and the mother (Fagan and Barnett, 2003; Pekel Uludağlı, 2017, p. 77; Tamis-LaMonda and Cabrera, 2008, p. 603), and the existing social structure influences father involvement.

In addition, father involvement must be meaningful and legitimate. Father involvement should also define a privileged position for fathers within social power relationships. As Giddens (2005, p. 223) states, society is composed of structuring meaning, sovereignty, and power relations. The phenomenon of father involvement can only reach the desired level when it finds its place within this relational space, and when social approval is valid and strong. Thus, father involvement can internalize themselves as part of their lived experiences within the lifeworld. How does an earthquake affect father involvement?

### 2.3 Post-earthquake father involvement

The destruction and trauma caused by the “disaster of the century” have affected families as deeply as individuals. The loss of family members, property, the fear of the future following the earthquake can lead to mental problems such as depression in mothers, fathers, and children. While adult family members must strive to protect their mental health, they are also responsible for supporting their children. Earthquakes can lead to changes in relationships and interactions between family members. Weitzman and Behrman (2016, p. 185) found that earthquakes increase tensions within families, leading to an increase in intimate partner violence (IPV). Lowe et al. (2012) examined the impact of Hurricane Katrina on close relationships and found that while most participants experienced negative changes in their relationships, some reported that their relationships became stronger despite the tension. Tekin Babuç and Gökçe (2024) stated that disasters can lead to changes in people’s identities and social networks. Their study emphasized that in the post-disaster period, families often take on new roles and responsibilities. Yanardağ (2024) indicated that the education and psychosocial well-being of earthquake-surviving adolescents aged 12–19 living in container cities were negatively affected due to the inadequate private living spaces, technological resources, social areas, and study environments. İn addition, limited privacy and living spaces have led to difficulties in managing emotions and fulfilling the need for solitude. Sevim (2024) stated that shifts have occurred in the roles of fathers within earthquake-affected families and that the surrounding environment plays a significant role in shaping the direction of these changes.

After disasters such as earthquakes, the way fathers form relationships with family members is influenced by pre-existing mother–father-child dynamics and parents’ post-event attitudes. According to Proctor et al. (2007), young children’s responses to environmental disasters are influenced by the quality of the parent–child relationship and parental behaviors following the event. Their research showed that parental stress significantly affects children’s psychological distress, while positive parental behaviors can mitigate these effects (Proctor et al., 2007). Psychological studies have revealed that post-earthquake stress disorder occurs and that disasters lead to increased irritability in fathers, thereby having a greater impact on children (Kılıç et al., 2004). Kuru and Ungar (2025) demonstrated that the psychological well-being of children in disaster-affected families is significantly compromised when parental mental health is impaired. However, elevated parenting quality may attenuate this risk. Within this framework, engaged fathering functions as a critical resilience-promoting factor that fosters children’s adaptive responses to post-disaster adversity.

Based on the literature mentioned above, this study seeks to answer the question of which factors influence father involvement in container cities after the earthquake, and how father involvement manifests within the context of these factors.

## 3 Methods

### 3.1 Research methodology

This study adopts hermeneutic phenomenology as developed by van Manen (2022) to explore the essence of lived experience. This approach aims to uncover the meaning of any given experience and its impact on the individual in a deep and reflective manner (Alsaigh and Coyne, 2021; Moley et al., 2018; van Manen, 2016; Wentz, 2005). Van Manen’s method involves six procedural activities: (1) formulating a research question); (2) investigating experience as it is lived, rather than conceptualized; (3) reflecting on essential themes that characterize the phenomenon; (4) describing the phenomenon through the process of writing and rewriting; (5) maintaining a strong and pedagogical orientation toward the phenomenon; and (6) balancing the research context by considering the parts and the whole (van Manen, 2016, p. 31).

In this study, the research problem was clearly articulated to identify the factors influencing father involvement among earthquake-affected fathers and to understand the current state of their involvement. One of the authors, himself the father of two who experienced the earthquake firsthand, brought an insider perspective that enabled both experiential engagement and a critical problematization of the issue. Thus, the first two steps of Van Manen’s framework were meaningfully addressed within the study design.

The literature review conducted by the authors indicated that the factors affecting father involvement may diverge from those previously identified in the literature. In-depth interview data further supported this observation. This article has undergone multiple rounds of writing and rewriting to reach its current form -an evolving process that could have taken different directions had it continued. In conclusion, it is evident that the authors remained committed to phenomenological reflection throughout the research process and attempted to construct interpretations in line with this orientation. While the trustworthiness of this approach ultimately rests on the reader’s judgment, the procedures undertaken to ensure this are outlined below.

### 3.2 Interview development, procedures and implementation

Building on a focused review of the relevant literature, we developed a semi-structured interview protocol to explore father involvement among earthquake-affected fathers through in-depth interviews. To assess question clarity and flow, we ran a small pilot study with three earthquake-affected fathers, during which four interviewers (two of whom were study authors) jointly conducted the sessions. The interview team consisted of two sociologists (professor and associate professor), one psychologist (assistant professor), and one associate professor specializing in educational measurement and evaluation. This procedure calibrated the interview team and enabled revisions based on direct observations. The audio was recorded and the pilot interviews were transcribed and reviewed solely for refining the instrument; no pilot data were included in the analysis. The pilot interviews were transcribed, and based on identified gaps or ambiguities, necessary revisions were made to finalize the semi-structured interview guide (see Supplementary Information 1). To further explore the participants’ lived experiences in depth, probing questions were employed during the interviews.

In accordance with ethical standards, both verbal and written informed consent were obtained from all participants prior to data collection to prevent any ethical concerns (see Supplementary Information 2). The study was approved by the Ethics Committee of Kilis 7 Aralık University (Decision No. 09, October 13, 2023) (see Supplementary Information 3). Legal research permissions were obtained from the Governorships of Hatay and Kahramanmaraş (see Supplementary Information 4). This study was designed, conducted, and reported in accordance with the COREQ checklist (Tong et al., 2007); the completed checklist indicates the page location of each item within the manuscript (see Supplementary Information 5).

In-depth interviews were conducted with 23 earthquake-affected fathers, including 13 from Hatay and 10 from Kahramanmaraş. The interviews were conducted in May 2024, with durations ranging from 45 to 82 min. With the participants’ consent, all interviews were audio-recorded and transcribed verbatim. These transcripts were first reviewed by the authors and then imported into MAXQDA 2020 for qualitative analysis. All data were securely stored on the authors’ institutional computers. Transcripts were not returned to participants for comment or correction. Participants did not provide feedback on the study findings.

### 3.3 Sampling

Purposive sampling was used in this study. This sampling strategy is commonly used in in-depth qualitative research, as it enables the deliberate selection of information-rich cases that can provide meaningful insights into the research questions (Ahmad and Wilkins, 2024; Creswell, 2013; Patton, 2015). Among the various purposive sampling strategies, we adopted *criterion sampling* (Patton, 2015, p. 405). Participants were selected based on a set of predefined eligibility criteria, and in-depth interviews were conducted with those who met these criteria.

The inclusion criteria were as follows: (1) having personally experienced the Kahramanmaraş earthquakes, (2) being the father of at least one child between the ages of 3 and 6, (3) not having lost any children in the earthquake, (4) having lived in a container settlement for at least 6 months, and (5) currently residing with one’s spouse and children. Fathers meeting these criteria were identified and approached for participation in collaboration with social workers actively engaged in container settlements, who acted as gatekeepers. Although some individuals declined to participate for various reasons, in-depth interviews were conducted with the 23 fathers. In accordance with the phenomenological research approach, both data and theoretical saturation were reached. In other words, as the interviews began to yield repetitive statements and no new themes emerged, the number of participants was limited to 23. The sociodemographic data selected for these participants are presented below.

Table 1 clearly illustrates that the participants had low levels of education and income. The number of children ranged from one to four, while the fathers’ ages varied between 24 and 50. The duration of marriage among participants ranged from 4 to 19 years. Most participants came from socioeconomically disadvantaged backgrounds, with educational attainment typically limited to lower secondary or high school. The relatively low education levels of their spouses further indicate the persistence of traditional family role patterns. The majority are employed as manual laborers, while only a few hold positions as civil servants or teachers. Monthly earnings generally correspond to the minimum wage or at most twice the amount. Overall, these findings portray a participant group facing significant economic hardship and sociological vulnerability in the post-earthquake context.

**Table 1:** Sociodemographic characteristics of the participants (*N* = 23).

Participants	Interviewers	Age	Education level	Occupation	Marital duration (years)	Number of children	Monthly income	Province
P1	I-1	27	College graduate	Worker	4	1	Minimum wage	Hatay
P2	I-2	34	Lower secondary	Worker	11	3	Minimum wage X 2	Hatay
P3	I-1	31	College graduate	Civil servant	5	2	Minimum wage X 2	Kahramanmaraş
P4	I-3	43	High school	Worker	19	4	Minimum wage X 2	Kahramanmaraş
P5	I-4	32	High school	Worker	8	3	Day labor	Hatay
P6	I-4	41	High school	Worker	14	3	Minimum wage	Hatay
P7	I-3	31	High school	Civil servant	10	2	Minimum wage	Kahramanmaraş
P8	I-3	20	Lower secondary	Worker	5	2	Minimum wage	Hatay
P9	I-4	38	Primary education	Worker	9	2	Minimum wage	Hatay
P10	I-1	26	Lower secondary	Worker	5	2	Minimum wage	Hatay
P11	I-3	31	Primary education	Worker	9	3	Day labor	Kahramanmaraş
P12	I-4	33	Lower secondary	Worker	6	3	Minimum wage	Kahramanmaraş
P13	I-3	42	College graduate	Civil servant	10	2	Minimum wage X 2	Hatay
P14	I-1	35	Primary education	Worker	11	3	Minimum wage	Kahramanmaraş
P15	I-3	29	Lower secondary	Worker	8	2	Day labor	Hatay
P16	I-2	33	College graduate	Teacher	5	1	Minimum wage X 3	Kahramanmaraş
P17	I-3	29	Lower secondary	Worker	5	2	Minimum wage	Kahramanmaraş
P18	I-1	31	High school	Unemployed	5	2	Minimum wage	Hatay
P19	I-4	35	High school	Unemployed	5	1	non-wage	Hatay
P20	I-1	38	Lower secondary	Unemployed	13	3	Day labor	Hatay
P21	I-4	42	High school	Civil servant	8	2	Minimum wage X 2	Kahramanmaraş
P22	I-1	33	Lower secondary	Worker	9	1	Minimum wage	Hatay
P23	I-2	31	College graduate	Worker	6	1	Minimum wage X 2	Kahramanmaraş

In May 2024, the statutory net minimum wage of Türkiye was ₺20,002.

### 3.4 Data analysis and coding

During the analysis phase, reflection on the main themes and the writing and rewriting of those themes, which corresponded to the third and fourth research activities outlined by Van Manen, came to the forefront. Within this framework, the study identified the factors affecting father involvement in the post-earthquake context and revealed how the trajectory of father involvement unfolded, as understood through interpretive analysis.

One of the aims of the study was to interpret, from a phenomenological perspective, how fathers’ involvement in their children’s lives transformed within their subjective lived worlds **-**that is, how their paternal engagement took shape and shifted. For this reason, the data were analyzed within an inductive framework, with the goal of uncovering the relational dimensions of father involvement.

The study followed the analytical steps of hermeneutic phenomenology to ensure transparency and adherence to scientific rigor: (1) *initial coding*, (2) *reflective coding*, and (3) *thematic condensation*. These procedural steps can be described as the application of Van Manen’s holistic, selective, and line-by-line reading approaches, through which meaning units were developed (van Manen, 2016). Accordingly, in this study coding and theme development are reported in terms of these readings. This analytic process was conducted to illuminate the factors shaping fathers’ experiences of involvement in container cities and to understand the current state of father involvement. Each phase was carefully designed to reflect Van Manen’s phenomenological research approach, characterized by reflective depth, hermeneutic circle, and iterative writing.

**Figure 1:**
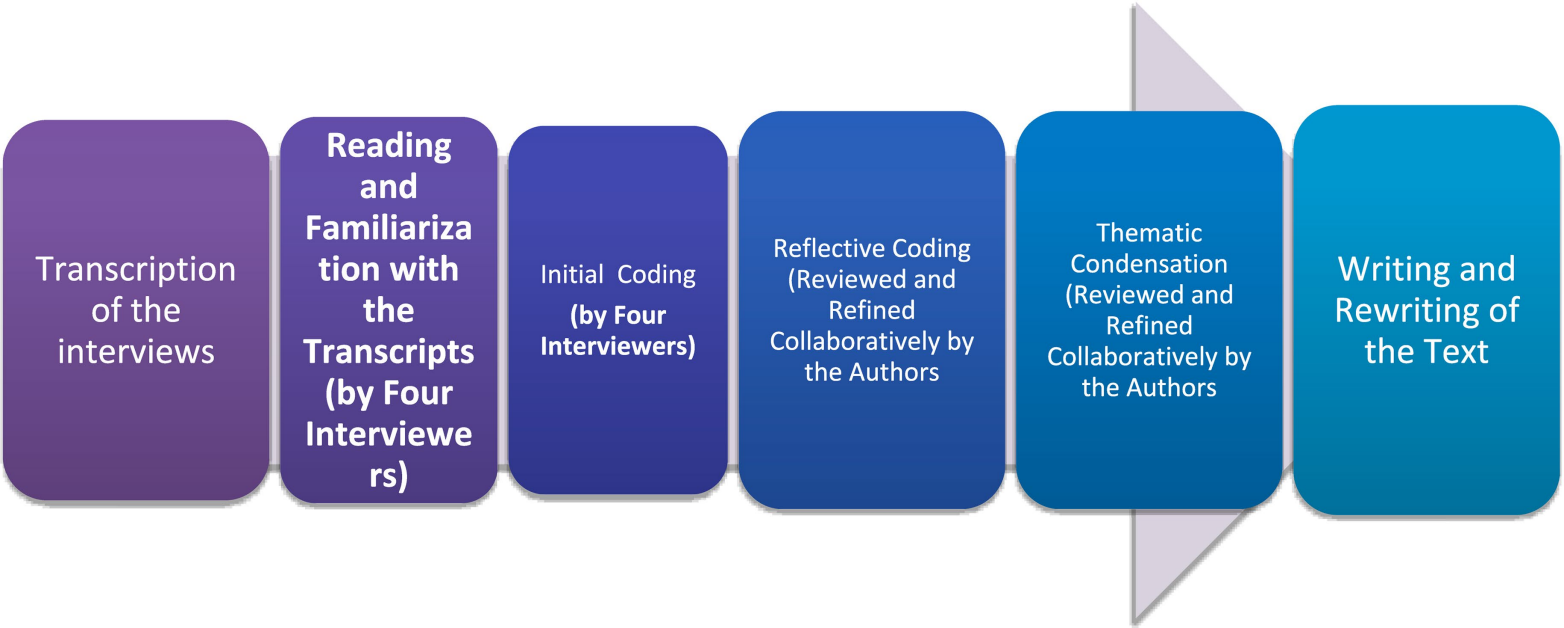
The analytic process.

Following the assignment of a code name to each participant (P1), the analytical process outlined in Figure 1 was implemented. During the initial coding phase, the interviewers read the transcripts and identified salient expressions, coding them in a manner that remained as faithful as possible to the participants’ wording and narrative style. Subsequently, we reviewed and discussed the codes collaboratively. They grouped codes reflecting father involvement and their influencing factors based on their semantic similarities. As a result of this grouping process, during the thematic condensation process, several clusters of codes were found to point toward broader themes, which were then interpreted and presented in detail in the Results section.

### 3.5 Trustworthiness

In qualitative research, certain trustworthiness criteria must be observed: credibility, dependability, confirmability, and transferability (Lincoln and Guba, 1985). In this study, every effort was made to adhere to these criteria. Given that the methodological framework is hermeneutic phenomenology, particular attention was paid to ensuring consistency, transparency, and auditability in both data collection and interpretation processes (van Manen, 2016). To enhance the first two elements, credibility and dependability, interviews were fully transcribed verbatim and excerpts were presented as such in the findings section. Additionally, both initial and reflective coding were performed through collaborative negotiation. Records of these deliberative sessions were maintained to ensure audit trials and accountability. Coding was conducted at intervals to allow for analytical distance and reflection. Throughout the analysis, a hermeneutic cycle of writing and rewriting was maintained. The procedural steps undertaken during this iterative process are summarized in Figure 1.

In line with the remaining two trustworthiness criteria, confirmability and transferability, analytic memos were systematically written in MAXQDA to make potential researcher biases visible during both coding and theme development processes. This reflexive documentation enhanced transparency and analytical rigor. Furthermore, the descriptive excerpts presented in the findings section, embedded within the research context, facilitate transferability by allowing readers to assess the applicability of interpretations to other settings. Given the phenomenological nature of the study and the collaborative essence of qualitative inquiry, data generation is inherently shaped by dialogical negotiation among researchers. The frequent engagement in reflexivity and the maintenance of an audit trail by the authors further contribute to the trustworthiness of this study (Braun and Clarke, 2021; Lincoln and Guba, 1985).

### 3.6 Researcher background and reflexivity

This study was conducted by two male authors. The corresponding author, an associate professor of sociology specializing in family studies, lives in the earthquake-affected region of Türkiye and is a father of two. Having experienced earthquakes with his wife and children and believing that he narrowly escaped death, he brings profound contextual and emotional insight into the study. The second author is a professor in sociology whose expertise lies in identity, migration, and civil society, and he is the father of three. Both authors have a long-standing scholarly and personal engagement in family research. Their complementary expertise and distinct life experiences enhanced the interpretive depth and overall trustworthiness of this study.

## 4 Results

### 4.1 Factors influencing father involvement in container cities

**Figure 2:**
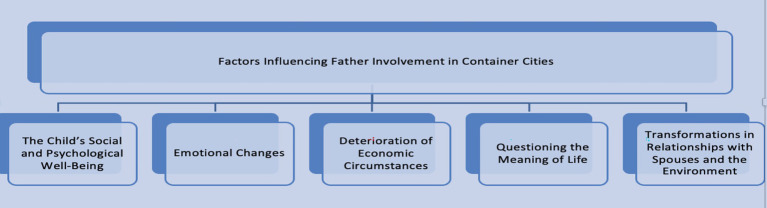
Factors influencing paternal involvement following the earthquake. The figure were created by the authors from the study’s data.

Hawkins and Palkovitz (1999) emphasized that the factors influencing father involvement are multidimensional and often overlooked because of measurement limitations. Therefore, they recommended higher sensitivity in measuring father involvement and emphasized their multidimensional nature. Changes in these influencing factors can be expected during the period following the earthquake. Following their advice, this study found that new circumstances arising from the earthquake influenced father involvement. The factors affecting father involvement among those living in container cities are shown in Figure 2.

These new factors have redefined the meaning of father involvement in container cities. Collectively, these findings underscore that father involvement is a sociological phenomenon.

#### 4.1.1 The child’s social and psychological well-being

Children’s physical and mental well-being, the presence of disabilities, and the success or failure of early attachment processes significantly influence father involvement. Fathers who maintain “strong father-child relationships” tend to experience less stress and invest more mental energy in their children (Li et al., 2024, p. 2303). The findings of this study were also consistent with those of Li et al. (2024). The situation of children who have witnessed many deaths, whose life-worlds have been displaced, and whose stocks of knowledge have become invalid has legitimized father involvement in fathers.

A father’s behavior toward involvement is directly linked to the child’s social and psychological conditions. As the earthquake altered children’s social and psychological conditions, father involvement also changed.

*“We give them medicine even for the slightest fever. An allergy developed afterward. Strange illnesses started popping up. House dust allergy has olso emerged. They still have little fear, like night terrors, do. We bring them to bed with us, that kind of thing.” (P1, Hatay)*

The earthquake led to frequent illnesses, the emergence of new health issues, and the development of night terror among children. Observing these changes, P1 adjusted his behavior, assumed responsibilities, and became more accessible to address his child’s needs.

Since earthquakes have deeply affect the psychological well-being of children, father involvement is important for children to remedy. For instance, children who are afraid of the dark, stutter, are forced to use the toilet, or are fearful of everything is particularly prevalent in container cities

*When I turn off the light, my youngest daughter says, ‘Daddy, there’s going to be an earthquake.’ You know, when the earthquake occured, lights and electricity went out. It happened right then. Now, every time the lights go off, she says, ‘Daddy, there will be an earthquake.’ (P2, Hatay)*

*My daughter had not stammed much before. I talked to a psychologist, who suggested that it might be a residual trauma from the earthquake. (P3, Kahramanmaraş)*

*We have gotten used to it, but the kids fear the slightest thing right now. Sudden rain affects them, and let me put it that way. Even strong winds have unsettled them. (P4, Hatay)*

*The five-year-old child saw everything about the earthquake. Forgive me, but they cannot go to the toilet alone. (.) We removed him, talked to him, and tried to forget him. We said, ‘There’s no such thing as an earthquake.’ Thankfully, we got through this. However, you know that they were really shaken. He’d say things like, ‘Are you going to leave me?’ His mental state was completely shattered but regained that sense of trust again. (P5, Hatay)*

*As a family, like my spouse and all, we keep thinking, ‘What would we do if there’s another earthquake?’ And I say, ‘Son,’ I tell him, ‘This is a container. We’d just roll over, fix it, and set everything up again.’ This is how he felt reassured. (P6, Hatay)*

Fathers engage in various participatory behaviors to ensure their children’s well-being. They sought solutions to their children’s challenges, worked to rebuild their sense of trust, and engaged in activities to mitigate damage caused by the earthquake. These paternal involvements strengthen the bonds between fathers and their children.

The post-earthquake social and psychological conditions of children have multiplied the responsibilities of their fathers. Fathers’ efforts to strengthen their children’s well-being, their instilling of confidence, their search for expert support, and their behaviors to erase the negative effects of difficult days on their children indicate father involvement. In other words, the altered social and psychological state of children in the aftermath of the earthquake triggered fathers to engage more deeply in their involvement.

### 4.2 Emotional changes

The earthquake has led to the disruption of ontological security owing to the loss of life and property. Furthermore, the tragic events that fathers witnessed during the disaster affected them emotionally. The analysis revealed that changes in fathers’ emotional conditions significantly influenced their involvement with their children.

All participants reported losing a relative, their home, or another property. Some fathers also encountered cases where children or entire families had perished, which heightened their sense of protectiveness toward their own children.

*So, I started paying more attention to him, I became focused more on him, and I started being more involved because he was also very scared. He had stayed outside for several days. He subsequently developed pneumonia. Now, if he gets even the slightest flu, his lungs are immediately stained. (P1, Hatay)*

*I mean, seeing those corpses -fathers holding their children as they died together really affected me. After seeing that, I started to hold my children closer. (P8, Hatay)*

*Following the earthquake, a special attachment was developed. God forbid anything happened to them; it would have been much worse for us. (P9, Hatay)*

*After an earthquake, it is almost inevitable, like a sense of protectiveness. After the earthquake, even in the container, I did not have the courage to leave them in another room in the container. (P10, Hatay)*

The death and suffering fathers witnessed during the earthquake pushed them to form stronger attachments to their children. It has been observed that fathers have engaged with their children more, paying greater attention to them and avoiding physical distancing.

The earthquake instilled a fear of “something might happen to their children” in fathers and turned them into “anxious father.”

*I worked with this man for six months, hardly ever coming home. But after the earthquake, I’ve never left my kids alone. (P11, Kahramanmaraş)*

*Since the earthquake, we have instinctively protected them. Wherever they go, I accompany them now. (P12, Kahramanmaraş)*

*I spent the last year and a half at home without interruption. (.) Right now, I do not see any places as safe. I go to pick up my child from school. He could go on his own, but I don’t trust that environment. (P13, Kahramanmaraş)*

*After the earthquake, I thought, ‘What if something happens to my kids?’ That is I became more protective. (P14, Kahramanmaraş)*

*We saw it, we saw the things that were shattered. We pulled out the babies’ bodies and staff. In those moments, I thought, ‘This could have been my child’. Sometimes it still comes to my mind, it even shows up in my dreams. (P17, Kahramanmaraş)*

The perceived “unreliability” of container cities, combined with the fear of harm coming to their children, has pushed fathers toward more protective behavior. Simultaneously, traumatic experiences of the earthquake deeply shook fathers, making them more attached to their children. They become more concerned about their children, spent more time with them, and engaged more in their care.

Post-earthquake uncertainties compelled fathers to navigate heightened emotions, resulting in challenges in regulating their behavior toward their children.

*Stress increases because of financial struggles. Kids want to spend time with you, but I sit and think about what to do next. At the same time, I know I need to spend time with them. Sometimes, I tell them, ‘Go play with this while I take a break.’ (P15, Hatay)*

*Before the earthquake, we were calmer, but now we are more irritable. Sometimes something happens, and you end up hitting your children. I definitely did not like this. At that moment, you act out of anger and hit them, and then you feel regret. (P8, Hatay)*

*I have been more short-tempered since the earthquake. I quickly lose patience. I did not like this before. (P5, Kahramanmaraş)*

After the earthquake, fathers faced difficulties in managing their emotions because of uncertainties in their new lives. This emotional turmoil sometimes manifests as violence, anger, or neglect toward their children.

A father’s emotions and thoughts toward his child determine father involvement (Diniz et al., 2021). The results of the analyses show that changes in father’s emotional conditions affect their involvement behaviors in two ways. On one hand, they drove fathers to be more protective and engaged, while on the other, they sometimes led to aggression or detachment. Excessive protective behaviors and loss of behavioral control toward their children can be considered risky or pathological in the context of the father-child relationship. However, the fact that fathers increasingly prioritize their children represents positive development. Their efforts to overcome the challenges and adversities they face also indicate an increase in father involvement.

#### 4.2.1 Deterioration of economic circumstances

In earthquake-affected regions, where fatherhood is often understood as bringing bread home and meeting the needs of family members, economic problems have significantly influenced father involvement. According to government reports, the economic cost of the earthquake was estimated to be $103.6 billion (Presidency of the Republic of Türkiye, Directorate of Strategy and Budget, 2024, p. 6). This economic loss has directly affected fathers living in earthquake-affected regions, leading to a transformation in their involvement.

The earthquake destroyed convertible assets, household belongings, and digital tools. This has resulted in economic instability and impoverishment of fathers, who are typically responsible for the household economy.

*After the earthquake, we faced significant hardship (.)Suddenly, it was gone. (P20, Hatay)*

*We have movies, phones, tablets, and computers. Everyone in the family has what they want. We had two televisions in separate rooms. (P9, Kahramanmaraş)*

*I have been married for eight years. Of course, in most families, life is equally shared. We should share everything, but first, I need to work to meet our basic needs. I need to bring money home. (P15, Hatay)*

Fathers who lost all their savings remembered the former condition as beautiful and good no longer existed. Moreover, accumulated debts and financial hardship led the father to experience intense stress, resulting in feelings of inadequacy. As a result of all these factors, fathers have questioned the meaning of fatherhood. In other words, the belief that “a person who cannot care for their children cannot be a father” has developed among fathers. This directly reflects on father involvement.

#### 4.2.2 Questioning the meaning of life

Suna Dağ et al. (2024), in their study on the effects of the February 6 earthquakes on families with amputee children, described the disaster as an “apocalypse” for parents, resulting in expressions of anger and frustration in its aftermath. Fathers who survived this “apocalypse” have begun reflecting on the question, *“What truly matters?”*

*I had a friend and I told him this. He is still working out of town and far away. He even lost his parents after the earthquake. I told him, ‘There are jobs you can do here as well. Be there for your children; hold on to them more closely.’ ‘That night, even the jacket on your back wasn’t yours.’ (P8, Hatay)*

*Many children died, as did many young people. We are among the lucky ones. We’ve been given another opportunity, and we need to make the most of it. (P21, Kahramanmaraş)*

The earthquake equalized all victims in terms of their disadvantages. Regardless of whether they were rich or poor, all were forced to endure the streets, cold, and profound pain. Fathers come back from the brink of death, realizing the need to make the most of this second chance. They increasingly rejected the notion that being away from their children to earn money holds much value, realizing that what truly matters is being present and becoming a better father.

The desire to live, arising from returning from the brink of death, has led to the removal of restrictions imposed on children. The role of the strict or disciplinary father has been replaced by that of the permissive father, who believes in the approach of ‘let the horse die if it must for the sake of the barley.’”

*For example, I avoided giving my child anything artificially. I tried to feed them completely as natural food. But now, when we’re at the store and they ask for chocolate, I think about it. I say, ‘Sure, we shouldn’t feed them that, but what if another earthquake happens in a year? What if both us -God forbid- are trapped? Let them enjoy it, at least.’ (P16, Kahramanmaraş)*

*In the past, we used to say, save money, put it aside. We’ll do this for our child later. Now, I don’t think about this at all. I say, let’s eat, drink, and travel. (P22, Hatay)*

*“I don’t need a house anymore. I just want a place to stay, earn enough to take care of my children. That’s it. I want to provide them with a future, but most importantly, to ensure that their education meets their basic needs. (P15, Hatay)*

Fathers expressed that wealth and possessions wealth and possessions have little meaning in a mortal world. What truly matters is living in harmony with life and ensuring that children grow into educated individuals.

Fathers’ questioning of their accustomed ways of life reshaped their involvement. The drive to accumulate wealth, a core impulse of capitalism, has, at least temporarily, been displaced by the earthquake. The devastation they experienced motivated fathers to place their children and families at the center of their lives. This shift has directly influenced their involvement, increasing their interaction and sense of responsibility, strengthening the concept of father involvement.

#### 4.2.3 Transformations in relationships with spouses and the environment

When analyzed through Bronfenbrenner’s ecological framework, father involvement can be understood within three distinct layers: ontogenetic (the father’s personal life story), micro (the characteristics of children and the mother), and macro systems (social structures) (Kulik and Sadeh, 2015, p. 20). While the micro and macro layers did not emerge as a specific theme in this study, it was evident that both mother and the environment significantly influenced father involvement.

The earthquake transformed fathers’ relationships with their spouses, highlighting the importance of spousal support.

*Inevitably, we now complete each other’s deficiencies and move forward. Sometimes I bathe the children, sometimes I change their diapers, dress them. For instance, our eating habits have changed compared to before. The house’s cleanliness has also suffered. Like now, I came in and found the house messy the children had scattered things around, so I cleaned up. I also help with cooking now and then. (P8, Hatay)*

Fathers’ increased time spent in the container and the limited space available compelled them to take on household responsibilities and participate in childcare. As a result, they began to provide more support to their spouses. However, the framing of their contributions as “helping” rather than sharing responsibilities equally indicates that an egalitarian family structure has yet to be fully reached.

After the earthquake, fathers began to worry about their children and wives, thinking about them, and becoming concerned about them. However, some participants noted that marital relationships had also been strained.

Post-earthquake, fathers began worrying about their spouses as well as their children. Nevertheless, some participants noted that their marital relationships were strained.

*For instance, I went with a friend to check an apartment. It was a concrete building. We stayed there for a while, but my wife said that she couldn’t handle it. She refused to stay in a concrete house. Even if I step outside, she immediately calls me. She is now more protective of me because of the earthquake. (P17, Kahramanmaraş)*

*For example, my wife still experienced psychological effects. I mean, how can I put this? It has an impact, it left its mark. (P19, Hatay)*

*We talk normally, but there’s still something missing in the husband-wife relationship. (P2, Hatay)*

The fathers reported that they were striving to improve the well-being of their spouses, who were negatively affected by the earthquake. For example, P19 avoided concrete buildings doe to his wife’s fear. In addition, it has been revealed that the physical condition of the container and limited living space negatively affect the relationship between spouses.

Since the environment is also undergoing transformation in the container city, father involvement is influenced by these changes.

*The entire neighborhood has changed. The same environment no longer exists. My old neighbors, my memories, the 30 years of life we built -it’s all gone. (P20, Hatay)*

*We used to have a safe home, the neighborhood was familiar. I mean, at worst, you know who might harm you. (.) But now, what I’m doing is this: I leave work, I drop the car off at five, and by twenty after, I’m at home. (P13, Hatay)*

*I’m anxious because there are many types of people here. You don’t know what might happen. The life in a container city is different. (P19, Hatay)*

*Whether it’s a neighborhood, a container, or just a community, there are different people, and some I’ve never seen before. The moment you turn your back, you don’t know what they’re doing and, what kind of work they’re involved in. You can’t entrust your children to them. You can’t let your children play a game with them. (P7, Kahramanmaraş)*

After the earthquake, the environment in the container city almost ‘entirely’ unreliable. This prompted fathers to monitor their children closely and keep them under constant supervision. This also indicates that accessibility is meaningful for fathers. Therefore, fathers want to stay close to their children and be there whenever they are needed.

Fathers learn how to build relationships with their children, often influenced by their surroundings. According to a study by Mother–Child Education Foundation (AÇEV, 2017), 39% of fathers reported learning fatherhood from their environment, demonstrating the significant impact of social surroundings on parenting. Similarly, spousal support, or “gatekeeping,” is another factors shaping father involvement. In this study, fathers offered greater support to their spouses, participated in childcare, and remained accessible to their children due to difficulties in container living conditions.

### 4.3 Father involvement in container cities

As previously mentioned, the earthquake highlighted new factors influencing father involvement. Considering the emerging new factors, the current state of father involvement in container cities is discussed below. Although participation in childcare is not yet sufficient according to interviews, involvement in childcare is a way to legitimize the discourse of “supporting the spouse.” Furthermore, fathers were found to take on economic responsibilities for their children, show a tendency toward being engaged fathers, and express a willingness to participate in games and activities with their children. In addition, fathers have made efforts to establish communication with their children. The fathers’ display of these behaviors suggests that they are making significant strides in terms of father involvement.

**Figure 3:**
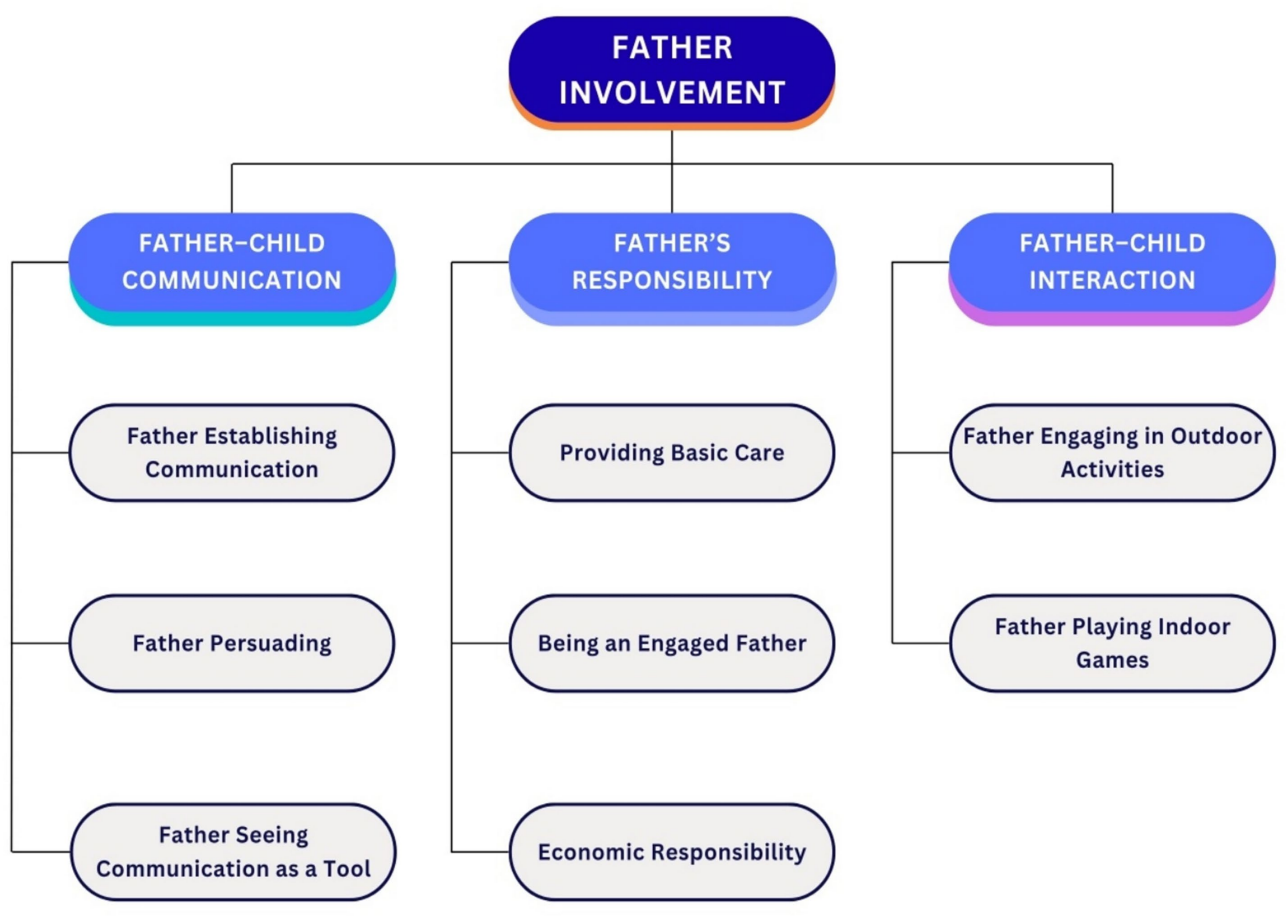
Father involvement in container cites. The figure was created by the authors from the study’s data.

As shown in Figure 3, the analysis of interviews revealed that father involvement focused on three themes: (1) communication, (2) interaction, and (3) responsibility. In the container city, fathers’ efforts to communicate with their children, persuade them, and establish a connection based on their belief in the power of communication indicates a significant transformation in their approach to parenting. In summary, they prioritize communication to protect and raise children in a new environment.

Fathers, by communicating with their children, not only get to know their children better but also engage with them more deeply. On the other hand, they strengthen the child’s bond with the father, which reinforces the need for increased father involvement.

*I’ve become more affectionate. After witnessing these things, I have changed me a lot. I try my best not to get angry anymore. (P14, Kahramanmaraş)*

*Compared to before the earthquake, my responsibility and communication with my children increased. (P12, Kahramanmaraş)*

*Sometimes my wife takes care of the younger child, and I take my older son out. For instance, we go to the market, or if there’s work at my grandfather’s house, I take him to help. Since he’s always with me. We inevitably have conversations. (P15, Hatay)*

P14, who previously portrayed a strict father figure, become softer and better understood his children after the earthquake. He acknowledged the importance of healthy communication with his children following traumatic experiences of the disaster. In addition, K15 stated that, since spending more time with his child after the earthquake, he has established mutual communication, that is, dialogue. This indicates that father involvement is meaningful.

For fathers living in a container city, responsibility as one of the key components of father involvement is, indicated through three sub-themes: basic care, engaged fatherhood, and meeting their children’s needs. Fulfilling these responsibilities demonstrated their active involvement in fatherhood.

It can be said that fathers prioritize their children’s responsibilities because they are concerned about their future. They shifted away from leaving all their responsibilities to their spouses.

*Right now, my focus is on creating a comfortable space for them, an environment where they can feel at ease. This is always in my mind. (..) Previously, I worked out of town all the time. But now, I’ve stopped doing so. I want to spend more time with them, to be more involved (P8, Hatay)*

*I don’t go to work. Honestly, I can’t leave the child and go anywhere, even though there are plenty of opportunities. But I just can’t leave my child behind. (P11, Kahramanmaraş)*

Because of the perception of the “unsafe” nature of container cities, fathers seek to remove their children from these settings and improve their living conditions, even slightly. To minimize safety concerns, fathers strive to stay close to their children, ensuring accessibility and care. Thus, they are accessible to their children and engage with them.

After the earthquake, fathers placed their children at the center of their lives, which made them prioritize spending time and engaging with them more.

*Before, my wife handled everything, including day care. Now it’s me. I go to school, talk to the teacher, and handle everything for school. (P10, Hatay)*

*For example, in the morning, my wife makes breakfast while I take care of the children watching TV, playing games and taking them outside. When it’s nap time, I put the baby to sleep, take the older one to preschool, and then bring it back. When they’re home, we play a little and talk about their days at school. I ask if they’re full and tell me about their day. (P5, Hatay)*

Fathers who had previously delegated all childcare responsibilities to their mother began actively following their children’s education and, to some extent, involvement in caregiving activities after the earthquake.

After the earthquake, fathers began to engage with their children out of need. To mitigate the negative effects of the earthquake, they started engaging in activities with their children both at home and outside.

*In other words, we used to play games before the earthquake, but we couldn’t take them on trips because of financial constraints. However, they need to be removed. For example, we attempted to take them to a village. (P6, Hatay)*

*After the earthquake, I started spending a slightly more time with them. There’s little work right now, so I have more time. (..) Sometimes we go to a park or a roadside area, or we play soccer. (P15, Hatay)*

*We’ve started playing more, and our bonds have been strengthened. (P23, Kahramanmaraş)*

Although fathers may agree to engage in outdoor activities with their children without spending time at home, these activities enable the transfer of social capital between fathers and children. The act of transferring social capital to one’s child can also be interpreted as an important indicator of father involvement.

*After the earthquake, I began spending more time on them. The more time I spent, the more I focused on them. (..) Yes, as I started spending more time, it became my priority. (P1, Hatay)*

*When I pick up a gun, my daughter immediately puts on her boots and says, ‘I’m coming with you.’ For instance, when we go quail hunting, I shoot the bird, but never pick it up. She always performs it herself. (P11, Kahramanmaraş)*

Increased time spent at home naturally enhances father involvement. As fatherhood is a cultural phenomenon, practices are reproduced based on circumstances. Fathers’ increased time spent with children and participation in activities indicate active involvement in fathering.

Through communication, responsibility, and interaction, fathers actively exhibit behaviors of involvement. Post-earthquake, these behavioral patterns have played a critical role in helping children cope with their new circumstances. In short, fathers have not left their children alone during challenging times; instead, they hold on to them and support them as much as possible.

## 5 Discussion

This study highlights the factors influencing father involvement in container cities and the current state of father involvement, offering significant contributions and innovations to the literature. (a) First, the study identified five factors influencing the father involvement in earthquake-affected fathers with children aged 3–6 identified: the child’s social and psychological condition, changes in emotional state, deterioration of economic circumstances, questioning the meaning of life, and transformations in relationships with the spouse and the surrounding environment. (b) Second, father involvement is performed in the forms of responsibility, interaction, and communication.

According to the findings, (a) first, father involvement is a sociological phenomenon and is performed when it is deemed “meaningful” for fathers. In short, father involvement is *context-dependent*. (b) Second, contrary to the expectation that the father-child relationship would weaken after the earthquake, it was revealed that fathers demonstrated father involvement behaviors. This suggests that father involvement is seen as legitimate and necessary by those living in container cities. (c) Finally, external pressures compelled fathers to engage in “solidarity” with family members, thereby increasing their involvement.

### 5.1 Father involvement is context-dependent

De Santis and Barham (2017, p. 960) classified the factors influencing father involvement as follows: father’s education status, average working hours, marital status, age, co-resident with child, occupation, quality of the marital relationship, emotional support during pregnancy, mental health, adherence to cultural norms, ability to fulfill economic responsibilities, father’s approach toward pregnancy, support to mother during pregnancy, mother’s socio-demographic background, mother’s work status, degree of shared parenting within the parental relationship, and mutual agreement between parents on parenting. Other researchers have proposed alternative classifications, such as (1) individual factors, (2) familial factors, (3) external family support, and (4) cultural influences (Diniz et al., 2021, p. 84). These factors influence father involvement across different social contexts and parent–child combinations.

The extraordinary conditions caused by the earthquake introduced new factors to this list, revealing that “context” plays a decisive role in father involvement. For example, Lamb’s three-dimensional model of father involvement- responsibility, accessibility, and interaction- evolved post-earthquake toward Palkovitz’s (2002, 2010) cognitive, affective, and behavioral models. The themes of questioning the meaning of life and emotional transformation demonstrate this evolution.

Indicators of the contextual nature of father involvement can be found in the “dynamic father involvement model” developed by Cabrera et al. (2007, p. 187). They stated that “contextual factors” (such as the mother–father relationship, economy, time, social ties, work, and religious activities) determine father involvement. In 2014, Cabrera et al. synthesized Belsky’s (1984, p. 83) *process model* with a dynamic model to propose an *expanded model*. In this model, each component was designed in a dialectical manner. According to them, fathers have a broader function in family life than is commonly believed. The sociological emphasis of Cabrera et al. aligns with the findings of this study, which suggests that fathers play a significant role in repairing the damage caused by earthquakes. In fact, the burden of addressing this damage may have been entirely placed on fathers’ shoulders. In this context, the earthquake can be considered a “contextual” factor that significantly influenced father involvement among those living in container cities.

### 5.2 Even deeper father-child attachment

Cabrera et al. (2007, p. 187) demonstrated that the characteristics of children -such as age, gender, disability, and temperament- significantly influence father involvement. Similarly, this study found that children’s social and psychological conditions emerged as key factors influencing father involvement. However, this factor does not fully align with Cabrera et al.’s (2007) model for two main reasons. (a) The first factor is related to fathers’ approach to mitigating the damage caused by the earthquake during the post-earthquake period. Having witnessed numerous families perish “holding each other,” fathers came to see their children as the only source of meaning and solace in life. This experience led fathers to view their children’s central focus of life, which made their existence meaningful. As a result, they attached more to their children, devoted themselves to them, and reoriented their lives around their children’s well-being. (b) The social and psychological conditions of children post-earthquake (such as disabilities, disadvantageous positions, and difficult or easy temperaments) created a demand for father involvement. In other words, the children’s circumstances necessitate fathers’ active involvement. For instance, research has shown that father involvement varies depending on whether children have difficult temperaments (Brown et al., 2011, p. 314). In container cities, children who have experienced an earthquake, endured psychological trauma, required therapy, faced displacement from their life worlds, and lost their social capital generate an even greater demand for father involvement. Consequently, fathers have intensified their efforts to protect their children by organizing activities, communicating more frequently, and, seeking professional support when necessary. These actions collectively contributed to greater father involvement.

### 5.3 Solidarity enhances father involvement

These findings suggest that solidarity increases father involvement, making a significant contribution to the literature. Solidarity with a spouse, extended family, or community directs fathers to participate in childcare. By supporting their spouses, fathers relieved some of their spouses’ burdens and began to participate in childcare and interactions with their children. Spousal support provided by fathers on both household chores and childcare helps mothers overcome the challenges posed by the earthquake. In addition, fathers have also been engaged in solidarity with their communities to organize activities for their children. These forms of solidarity foster father involvement, which in turn contributes to the well-being of children.

Previous studies have shown that spousal support produce positive outcomes. For instance, in their longitudinal study, Zhang et al. (2024) found that marital satisfaction positively influenced father involvement. Similarly, Kwok et al. (2013, p. 1057) demonstrated that marital satisfaction influences father involvement. Based on these findings, it can be concluded that marital satisfaction affects father involvement, and spousal support amplifies this effect. Thus, mutual support between spouses enhances marital satisfaction, which, in turn, positively impacts father involvement. In line with the findings of these studies, fathers’ support for their spouses in the aftermath of the earthquake may be interpreted as an indication of a “healthy” marital relationship, which in turn appears to have contributed to increased father involvement.

Beyond its role in promoting marital satisfaction, fathers’ involvement also appears to be derived from a strong desire to protect their children and spouses, ensuring that no issues remain unresolved as an indication of their commitment to safeguarding their families (Simonelli et al., 2016; Premberg et al., 2008, p. 231). Similarly, in container cities, fathers were observed to support mitigating the challenges faced by their children and spouses. This community effort has played a role in rehabilitating the well-being families during difficult post-disaster conditions.

## 6 Conclusion

This study examined the determinants of father involvement in the aftermath of a major earthquake. The findings revealed that father involvement is shaped by a range of dynamics, including the child’s social and psychological condition, changes in emotional state, deterioration of economic circumstances, questioning the meaning of life, and transformations in relationships with the spouse and the surrounding environment. These factors suggest that father involvement is not static but rather a context-dependent sociological phenomenon. The study further demonstrates that disaster conditions can lead to an even deeper father–child attachment, and that solidarity within the family and community plays a pivotal role in fostering father involvement. Ultimately, this study underscores the importance of strengthening father involvement as a key component of post-earthquake psychosocial recovery.

While negative outcomes among children in earthquake-affected families are expected, prioritizing efforts to enhance father involvement can help to remedy these impacts. In other words, initiatives should focus on fostering healthy father involvement in challenging environments such as container cities, camps, or tent settlements. To achieve this goal, an educational program needs to be developed to improve father involvement under extraordinary circumstances.

One of the key strengths of this study is its assertion that father involvement is sociological. However, the lack of comprehensive discussion on these claims in the existing literature necessitates further validation. Additionally, the increase in father involvement following a negative experience such as an earthquake may be influenced by pathological factors (anxiety about child loss, compensatory mechanisms for loss, etc.), which future studies should explore. It is important to remember that behaviors related to father involvement may primarily driven by safety concerns rather than a broader shift in parenting roles. In this context, further research supporting this study is crucial to understanding how the concept of father involvement develops under various conditions.

## Data Availability

The original contributions presented in the study are included in the article/Supplementary material, further inquiries can be directed to the corresponding author.
