# Supplementary material for: Enhancing father involvement of earthquake-affected fathers: a qualitative analysis
Source: Front Sociol. 2025 Nov 28;10:1657517. doi: 10.3389/fsoc.2025.1657517 (PMC12700030; doi:10.3389/fsoc.2025.1657517)
Supplement: Supplementary file 1 [file Supplementary_file_1.docx]

Pilot Interview: Interview Questions

1. **Could you please tell me a little about yourself?**
2. **Could you share the story of how you started your family?**
3. **In your view, what does it mean to be a father?**
4. **How did you learn to be a father?**
5. **How would you describe your relationship with your children?**
6. **How did the earthquake(s) affect your experience of fatherhood?**
7. **After the earthquake(s), how would you describe your relationship with your children? Did anything change?**

**Note:** Additionally, numerous follow-up probes were used.

**Interview Questions for the Main Study**

1. Please describe how you started your family.
2. What does fatherhood mean to you? *(How do you define being a father? Please provide examples.)*
3. Please characterize your communication and interaction with your child/children. *(Describe a typical day you spend together, with concrete examples.)*
4. In what ways does your spouse -or, if not applicable, your family/relatives- shape your relationship with your child/children? *(Please illustrate with examples.)*
5. Considering your broader environment (family, relatives, neighbors, neighborhood), what is expected of you as a father for your child/children? *(Please elaborate with examples.)*

- Is there anything you would like to add that we have not covered?

**Note**: Additionally, numerous non-leading follow-up probes were used to elicit depth and clarify meanings.
