# Supplementary material for: Enhancing father involvement of earthquake-affected fathers: a qualitative analysis
Source: Front Sociol. 2025 Nov 28;10:1657517. doi: 10.3389/fsoc.2025.1657517 (PMC12700030; doi:10.3389/fsoc.2025.1657517)
Supplement: Supplementary file 2 [file Supplementary_file_2.pdf]

T.C.
KİLİS 7 ARALIK ÜNİVERSİTESİ
ETİK KOMİSYONU

KATILIMCILAR İÇİN BİLGİLENDİRİLMİŞ GÖNÜLLÜ OLUR FORMU

Sizi, **Kilis 7 Aralık Üniversitesi Etik Komisyonu'**ndan ……………………………….tarih / ………………………………….sayı ile izin alınan* ve **Doç. Dr. Mehmet Fatih GÜLOĞLU** tarafından yürütülen **"Deprem Sonrasında Baba Katılımının İyileştirilmesi: Hatay ve Kahramanmaraş Örneği"** başlıklı araştırmaya davet ediyoruz. Bu çalışmaya katılmak tamamen gönüllülük esasına dayanmaktadır. Çalışmaya katılmama veya katıldıktan sonra herhangi bir anda çalışmadan çıkma hakkına sahipsiniz. Bu çalışmaya katılmanız için sizden herhangi bir ücret istenmeyecektir. Çalışmaya katıldığınız için size bir ödeme yapılmayacaktır. Çalışmadan elde edilecek bilgiler tamamen araştırma amacı ile kullanılacak olup kişisel bilgileriniz gizli tutulacaktır.

*Kilis 7 Aralık Üniversitesi Etik Komisyon izini alındıktan sonra doldurularak kullanılacaktır.

**Araştırmanın Adı**	Deprem Sonrasında Baba Katılımının İyileştirilmesi: Hatay ve Kahramanmaraş Örneği	
**Araştırmanın Amacı**	Bu proje çalışmasında aşamalı olarak deprem sonrasında baba katılımının nasıl anlamlandırıldığı ve ne düzeyde olduğunun ortaya konulması, depremzede babaların katılımına katkı sağlayabilecek bir eğitim modülünün etkiliğinin sınanması amaçlanmıştır	
**Araştırmanın Yöntemi**	Bu çalışma çok aşamalı karma yöntem araştırma olarak modellenmiştir. Çalışmanın ilk aşamasında nicel verilerin elde edileceği örneklem olarak Hatay'dan en az 258, Kahramanmaraş'tan 302 3-6 yaş çocuğu olan babanın tesadüfi seçilmesi planlanmıştır. Nitel veriler ise 20-30 gönüllü katılımcı babadan elde edilecektir. İkinci aşamada ise 20 deney, 20 kontrol olmak üzere 40 babayla bir deneysel çalışma yürütülmesi planlanmaktadır.	
**Araştırmanın Nedeni**	**Bilimsel araştırma**	
**Araştırmanın Öngörülen Süresi (Başlama ve Bitiş Tarihi)**	15.11.2023- 15.05.2025	
**Araştırmaya Katılması Beklenen Katılımcı/Gönüllü Sayısı**	**650 Katılımcı**	
**Araştırmanın Yapılacağı Yerler**	Hatay ve Kahramanmaraş il merkezleri	
**Görüntü ve/veya ses kaydı alınacak mı?**	☒ Evet	Hayır

Tablo katılımcıların anlayabileceği biçimde, akademik dil kullanılmadan yazılacaktır.

KATILIMCI BEYANI

Yukarıda amacı ve içeriği belirtilen bu araştırma ile ilgili bilgiler tarafıma aktarıldı. Bu bilgilerden sonra araştırmaya katılımcı olarak davet edildim. Bu çalışmaya katılmayı kabul ettiğim takdirde gerek araştırma yürütülürken gerekse yayımlandığında kimliğimin gizli tutulacağı konusunda güvence aldım. Bana ait verilerin kullanımına izin veriyorum. Araştırma sonuçlarının eğitim ve bilimsel amaçlarla kullanımı sırasında kişisel bilgilerimin dikkatle korunacağı konusunda bana yeterli güven verildi. Araştırmanın yürütülmesi sırasında herhangi bir sebep göstermeden çekilebilirim. Araştırma için yapılacak harcamalarla ilgili herhangi bir parasal sorumluluk altına girmiyorum. Bana herhangi bir ödeme yapılamayacaktır. Araştırma ile ilgili bana yapılan tüm açıklamaları ayrıntılarıyla anlamış bulunmaktayım. Bu çalışmaya hiçbir baskı altında kalmadan kendi bireysel onayım ile katılıyorum. İmzalı bu form kağıdının bir kopyası bana verilecektir.

Bu belge, güvenli elektronik imza ile imzalanmıştır.
