# Supplementary material for: Enhancing father involvement of earthquake-affected fathers: a qualitative analysis
Source: Front Sociol. 2025 Nov 28;10:1657517. doi: 10.3389/fsoc.2025.1657517 (PMC12700030; doi:10.3389/fsoc.2025.1657517)

# EK 3 (Etik Kurul Raporu)

Evrak Tarih ve Sayısı: 16.10.2023-E.36563


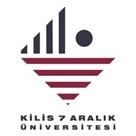
T.C.

KİLİS 7 ARALIK ÜNİVERSİTESİ REKTÖRLÜĞÜ

Genel Sekreterlik

Sayı : E-76062934-044-36563 16.10.2023

Konu : Etik Kurul Kararı (Doç. Dr. M. Fatih

GÜLOĞLU)

İNSAN VE TOPLUM BİLİMLERİ FAKÜLTESİ DEKANLIĞINA

Fakülteniz Öğretim Üyesi Doç. Dr. M. Fatih GÜLOĞLU'na ait çalışmanın uygun olduğuna dair etik kurul kararı ekte gönderilmiştir.

Bilgilerinize arz ederim.

Necdet BOZGEYİK

Genel Sekreter

Ek:Etik Kurul Kararı (1 Sayfa)

Bu belge, güvenli elektronik imza ile imzalanmıştır.

Belge Doğrulama Kodu :BSDZ8LY7S Belge Takip Adresi : https://turkiye.gov.tr/ebd?eK=6674&eD=BSDZ8LY7S&eS=36563

Adres:Mehmet Sanlı Mah. Doğan Güreş Paşa Bul. No:84 Kilis Telefon:+90 348 814 26 66 Faks:0 348-814 26 60

[e-Posta:ozelkalem@kilis.edu.tr](mailto:ozelkalem@kilis.edu.tr) We[b:https://www.kilis.edu.t](http://www.kilis.edu.tr/)r Kep [Adresi:kilis7aralikuniversit](mailto:kilis7aralikuniversitesi@hs01.kep.tr)[esi@hs01.kep.tr](mailto:esi@hs01.kep.tr)

Bilgi için: Rufeyde Gökçe


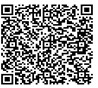
Unvanı: Şef

Tel No: 1066/1068


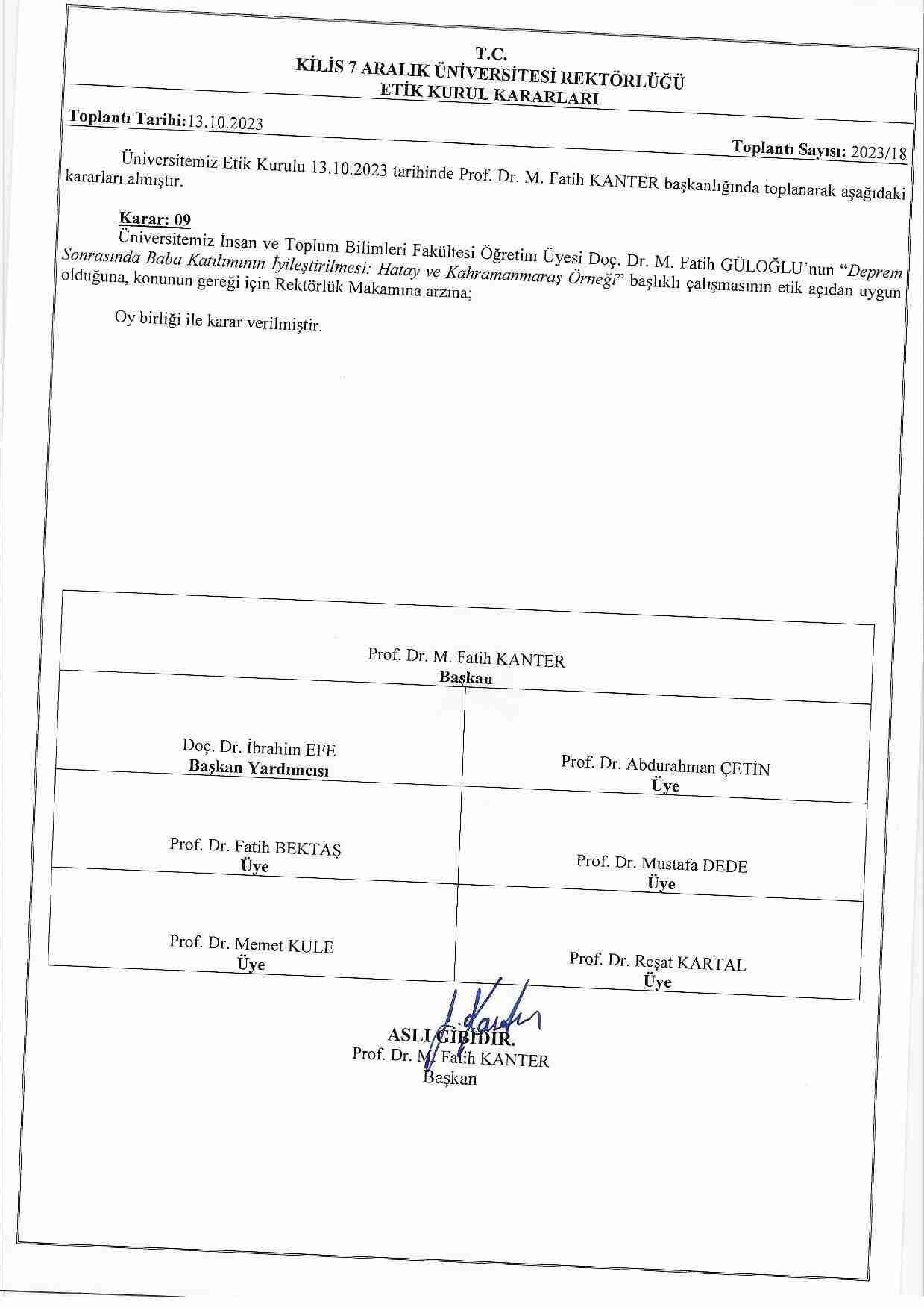

Supplement: Supplementary file 3 [file Supplementary_file_3.docx]
