# Supplementary material for: Enhancing father involvement of earthquake-affected fathers: a qualitative analysis
Source: Front Sociol. 2025 Nov 28;10:1657517. doi: 10.3389/fsoc.2025.1657517 (PMC12700030; doi:10.3389/fsoc.2025.1657517)
Supplement: Supplementary file 4 [file Supplementary_file_4.docx]

# EK 1 (İzinler)


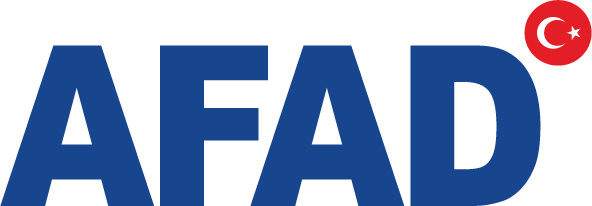

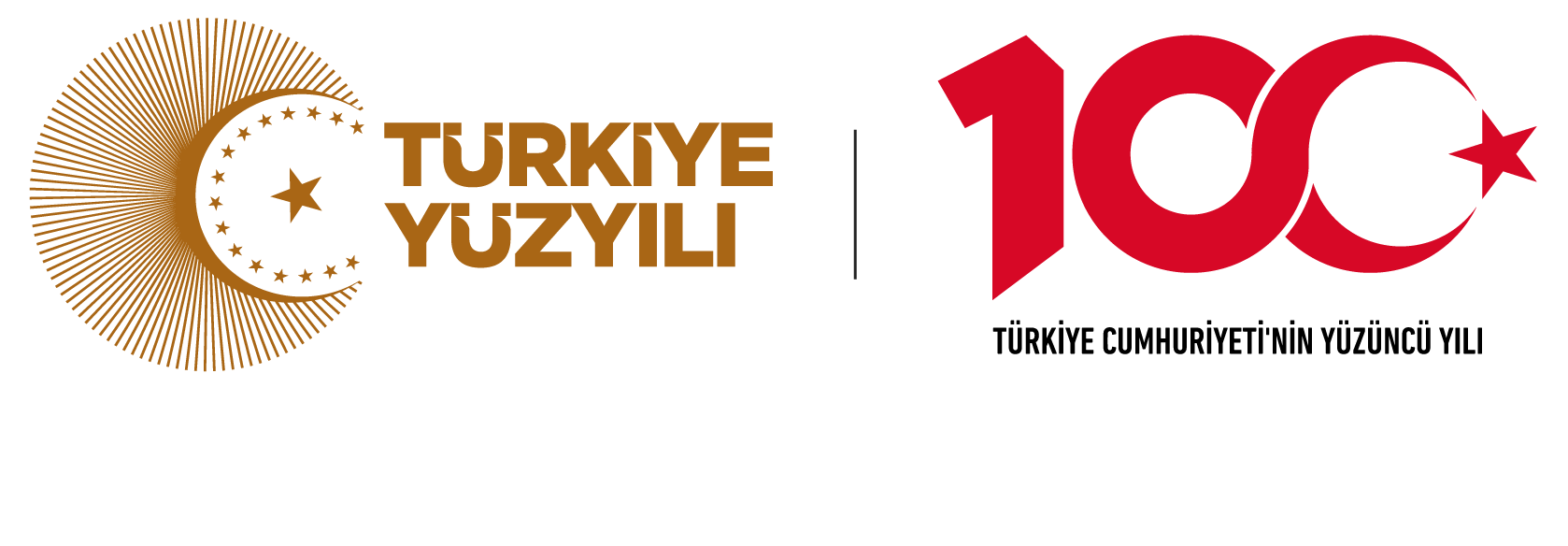
T.C.

HATAY VALİLİĞİ

İl Afet ve Acil Durum Müdürlüğü

Sayı : E-58360657-622.03-707393

Konu : Veri Talebi (Doç. Dr. Fatih GÜLOĞLU)

KİLİS 7 ARALIK ÜNİVERSİTESİ REKTÖRLÜĞÜNE

(Genel Sekreterlik)

İlgi : 20.09.2023 tarihli ve 76062934-605.01.01.01-E.34112 sayılı yazınız.

05.10.2023


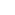
İlgi yazınızda belirtilen; Üniversiteniz İnsan ve Toplum Bilimleri Fakültesi Sosyoloji Bölümü öğretim üyelerinden Doç. Dr. Mehmet Fatih GÜLLÜOĞLU'nun "TUBİTAK 1001" programı ve "223K007" numaralı "Deprem Sonrasında Baba Katılımının İyileştirilmesi; Hatay ve Kahramanmaraş Örneği" adlı proje kapsamında İlimizde bulunan depremzedelerin barındığı konteyner yerleşkelerinde veri toplanması ve depremzede vatandaşlara eğitim verilmesi İl Müdürlüğümüzce uygun görülmüştür.


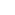
Bilgilerinizi ve gereğini arz ederim.

Ali İhsan KÖRPEŞ

İl Afet ve Acil Durum Müdürü

Bu belge, güvenli elektronik imza ile imzalanmıştır.

Doğrulama Kodu: 83589B66-871B-40B2-B39E-256E16894D96 Doğrulama Adresi: [https://www.turkiye.gov.tr/afad-ebys](http://www.turkiye.gov.tr/afad-ebys)

İSKENDERUN YOLU ÜZERİ 23.KM PAŞAKÖY KÖPRÜSÜ YANI TAVLA


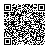


CADDESİ NO:1 ANTAKYA / HATAY Telefon No: (326) 233 54 15 Belge geçer No

: (326) 233 54 19

e-Posta: [hataymdr@afad.gov.tr](mailto:hataymdr@afad.gov.tr) İnternet Adresi: https://hatay.afad.gov.tr Kep Adresi: [hatayafad@hs01.kep.tr](mailto:hatayafad@hs01.kep.tr)

KEP Adresi : [hatayafad@hs01.kep.tr](mailto:hatayafad@hs01.kep.tr)

Bilgi için:Özhan ÖZGÜN

Şube Müdürü V. Telefon No:(326) 233 54 15


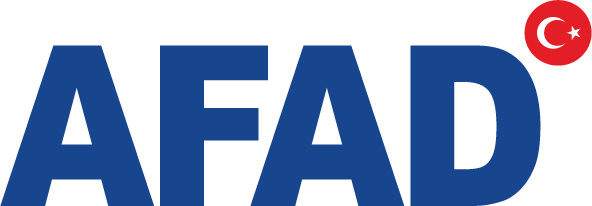

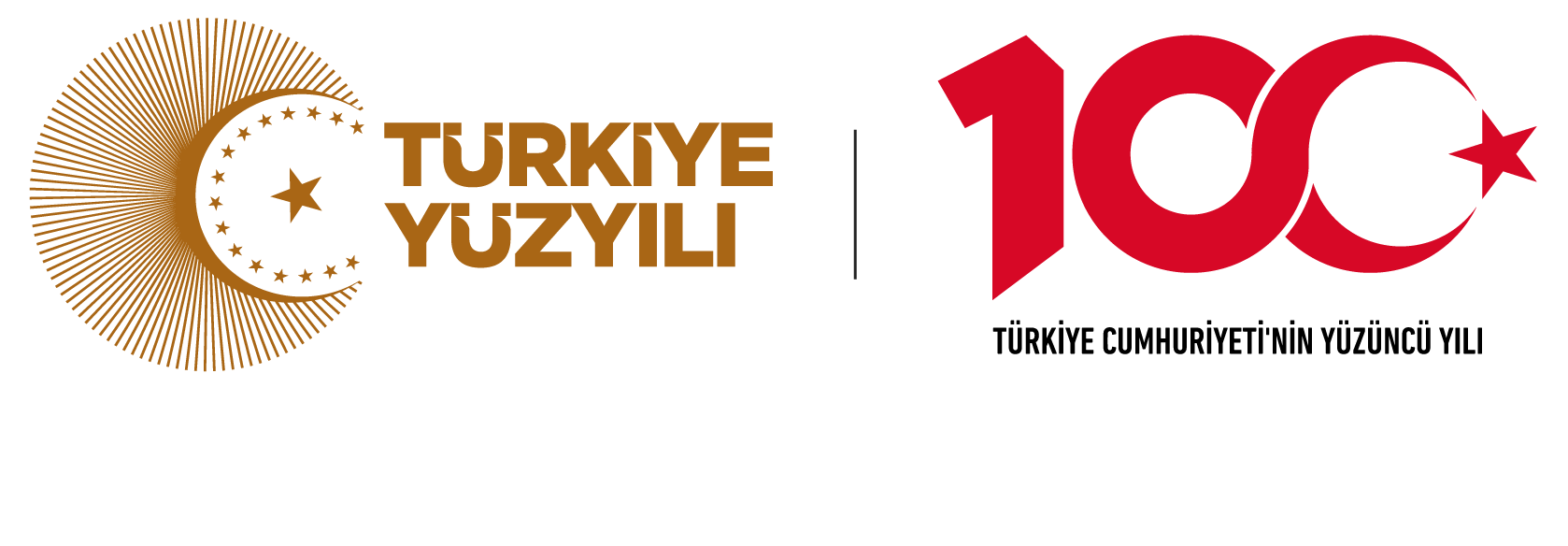


T.C.

KAHRAMANMARAŞ VALİLİĞİ

İl Afet ve Acil Durum Müdürlüğü

Sayı : E-48802187-622.03-707255

Konu : İzin Talebi

KİLİS 7 ARALIK ÜNİVERSİTESİ REKTÖRLÜĞÜNE

İlgi : 20.09.2023 tarihli ve 76062934-605.01.01.01-E.34113 sayılı yazınız.


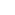
İlgi yazıda; Üniversiteniz İnsan ve Toplum Bilimleri Fakültesi Sosyoloji Bölümü öğreti̇m üyeleri̇nden Doç. Dr. Mehmet Fatih GÜLOĞLU'nun ''TÜBİTAK 1001'' programı ve ''223K007'' numaralı proje ile Kahramanmaraş ve Hatay illerinde bulunan depremzedelerin barındığı Afet ve Acil Durum Yönetimi (AFAD) başkanlığına bağlı olan konteyner yerleşkelerinde veri toplaması ve eğitim verebilmesine ilişkin kurumumuzdan yasal izin talebinde bulunmaktadırlar.


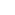
Bu kapsamda; Kahramanmaraş ilinde bulunan Afet ve Acil Durum Yönetimi (AFAD) başkanlığına bağlı olan konteyner yerleşkelerinde veri toplaması ve eğitim verebilmesi uygun görülmüştür.


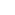
Bilgilerinize rica ederim.

Ekrem Ender ERGÜN

Vali a.

Vali Yardımcısı

Bu belge, güvenli elektronik imza ile imzalanmıştır.

Doğrulama Kodu: 07F9B402-A5CB-44F3-8CA5-4F49886542DA Doğrulama Adresi: [https://www.turkiye.gov.tr/afad-ebys](http://www.turkiye.gov.tr/afad-ebys)

Karacasu Mamaraş Mahallesi 84041 Sokak No:41 Dulkadiroğlu/ KAHRAMANMARAŞ Telefon No: (344) 224 14 14 Belge Geçer No: (344) 223 92 81


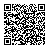


[e-Posta:kahramanmarasmdr@afad.gov.tr](mailto:kahramanmarasmdr@afad.gov.tr) İnternet

**Bu belg**A**e**d**,**r**g**es**ü**i:**v**ht**e**tp**n**s**l**:**i**//k**e**a**le**hr**k**a**t**m**ro**an**n**m**ik**ara**im**s.a**z**fa**a**d.**i**g**le**ov**i**.**m**tr **zalanmıştır.**

**Evrak s**K**o**e**r**p**g**A**u**d**la**re**m**si**a**: k**s**a**ı**h**h**ra**t**m**tp**a**s**nm**://**a**t**r**u**as**r**a**k**fa**iy**d**e**@**.g**hs**o**0**v**1.**.**k**t**e**r/**p**e**.t**b**r **d?eK=6674&eD=BSRZ5E4YS&eS**

KEP Adresi : [kahramanmarasafad@hs01.kep.tr](mailto:kahramanmarasafad@hs01.kep.tr)
